# Investigating the pH dependent antifungal effects of butyrate on *Candida albicans*

**DOI:** 10.3389/fmicb.2026.1793162

**Published:** 2026-03-23

**Authors:** Fiona C. Dresel, Campbell W. Gourlay

**Affiliations:** School of Natural Sciences, University of Kent, Canterbury, United Kingdom

**Keywords:** antifungal, butyrate, *Candida albicans*, dysbiosis, microbiome

## Abstract

**Introduction:**

*Candida* species are common members of the human gastrointestinal microbiome but are also associated with a range of diseases when microbial community balance is disrupted. Short-chain fatty acids produced by gut bacteria, particularly butyrate, play important roles in host–microbiome interactions and are increasingly explored as therapeutic modulators of microbial composition. Butyrate is present both as a microbial metabolite and dietary component and has been reported to influence Candida abundance within the gut. However, the antifungal activity of sodium butyrate (NaB) against *Candida albicans* under physiologically relevant gut pH conditions remains poorly understood.

**Methods:**

We examined the effects of NaB on *C. albicans* growth and physiology under pH conditions representative of the gastrointestinal environment. Growth, hyphal transition, respiration and biofilm formation were assessed in the presence of NaB at neutral pH. Parallel experiments at acidic pH (pH 4.0) evaluated fungicidal activity and associated cellular responses, including mitochondrial membrane potential, reactive oxygen species (ROS) accumulation, and intracellular calcium homeostasis. Histone deacetylase inhibitory activity of NaB was also assessed to determine its early cellular effects.

**Results:**

NaB displayed rapid histone deacetylase inhibitor activity in *C. albicans* and significantly inhibited growth, hyphal morphogenesis, respiration, and biofilm formation at neutral pH. In contrast, under acidic conditions (pH 4.0) NaB exhibited fungicidal activity. This lethal effect was associated with mitochondrial depolarisation, elevated ROS levels, and disruption of intracellular calcium regulation. Further analyses indicated that oxidative stress and loss of calcium homeostasis are key contributors to NaB-induced cell death under acidic conditions.

**Discussion:**

These findings reveal a strong pH dependence in the antifungal activity of butyrate against *C. albicans*. While NaB acts primarily as a physiological inhibitor of growth and virulence traits at neutral pH, acidic conditions convert its activity to a fungicidal mechanism driven by mitochondrial dysfunction, oxidative stress, and calcium dysregulation. This pH-dependent behaviour has implications for understanding microbiome-derived metabolites in fungal ecology within the gut and highlights the potential of butyrate-based strategies to modulate *C. albicans* overgrowth.

## Introduction

The composition of the gut microbiome is increasingly recognised as a critical factor in human health and disease. For example, in patients with neurodegenerative diseases such as amyotrophic lateral sclerosis, significant alterations in gut microbial diversity and metabolite production have been documented (Fang et al., 2016; Rowin et al., 2017; Hertzberg et al., 2022). The short chain fatty acid butyrate, produced by gut bacteria such as *Roseburia intestinalis* and *Eubacterium rectale,* is produced during microbial fermentation of dietary fibre (Trachsel et al., 2016). Butyrate serves as an energy source for colonocytes (Donohoe et al., 2011) and functions as a histone deacetylase (HDAC) inhibitor (Candido, 1978; Vidali et al., 1978; Donohoe et al., 2014; Pascale et al., 2018; Chen et al., 2019) with immunomodulatory and neuroprotective effects (den Besten et al., 2013). Butyrate has also been shown to exert neuroprotective functions via its HDAC inhibitor activity (Chen et al., 2019; Yan et al., 2024), to improve gut barrier function (Liu et al., 2018) and to be important in regulating fungal colonisation within the gut (Chen and Vitetta, 2020; Ricci et al., 2022; McCrory et al., 2024).

The role of butyrate in regulating fungal populations has been highlighted in studies that simulate dysbiosis in the human gastrointestinal tract. For example the antibiotic-induced reduction of butyrate producing bacteria led to *C. albicans* overgrowth (Marsaux et al., 2025). Specific butyrate producing species, such as *Intestinionas butyriciproducens*, have been negatively correlated with *C. albicans* abundance in the human gut (Delavy et al., 2023). In dysbiotic gut environments, *C. albicans* overgrowth can exacerbate inflammation and cause mucosal damage. This phenomenon has been linked to inflammatory bowel diseases such as Crohn’s and ulcerative colitis, where increased *Candida* abundance correlates with disease severity (Jawhara, 2022; Jangi et al., 2024). Additionally, *C. albicans* overgrowth has been linked to small intestinal fungal overgrowth, a condition that causes symptoms like bloating, abdominal pain, and diarrhoea, with symptom improvement observed following antifungal therapy (Soliman et al., 2025). Increased *C. albicans* abundance has also been observed in colorectal cancer tissue, suggesting a potential contributory role in tumour-associated inflammation (Kaźmierczak-Siedlecka et al., 2020). These findings highlight that fungal dysbiosis, including *Candida* overgrowth, may contribute to disease severity and progression. They also highlight the importance of maintaining butyrate-producing microbial communities as key regulators of gut health and fungal balance (Guinan et al., 2019).

*C. albicans* is a dimorphic fungus capable of switching between yeast and hyphal forms, a key determinant of its ability to colonise as a biofilm and to invade host tissues (Berman and Sudbery, 2002; Nobile et al., 2008). Hyphal morphogenesis is tightly regulated by transcription factors (Garbe et al., 2023; Lee et al., 2023; Luther et al., 2023) and chromatin modifications, including those regulated by HDAC enzymes (Lopes da Rosa and Kaufman, 2012; Garnaud et al., 2016). Among a range of environmental factors that can influence hyphal formation in the gut, butyrate has been shown as capable of influencing transcriptional networks involved in morphogenesis (Biswas et al., 2007; Zacchi et al., 2010; Nguyen et al., 2011).

The gastrointestinal tract exhibits pronounced pH variation, from acidic conditions in the stomach (pH 1–2), to pH 4 in the lower part of the stomach to near neutral environments in the colon (pH 7) and up to pH 8 in the ileum. SCFAs, including butyrate, can contribute to regional acidification with their protonated and ionised forms predominating under different pH conditions. Consequently gut pH and butyrate levels may act collectively to influence host health and regulate the growth of opportunistic organisms such as *C. albicans* (Duncan et al., 2009; Ballan et al., 2020; de Vos et al., 2022; Yamamura et al., 2023). However despite the clear potential for pH to modulate the antifungal effects of sodium butyrate against *C. albicans* this has not been investigated, We show that while NaB suppresses *C. albicans* growth, hyphal transition and biofilm growth at neutral pH, it has fungicidal effects under acidic conditions by disrupting mitochondrial function, elevating reactive oxygen species, and deregulating calcium homeostasis. These findings highlight the importance of gut pH in shaping butyrate-fungal interactions and contributes to our understanding of how it may be used as a therapeutic to regulate *Candida* overgrowth and improve gut health.

## Materials and methods

### Strains and culture conditions

*C. albicans* wild type or strains deleted for HDAC genes were cultured in YPD (2% glucose, 1% yeast extract, 2% peptone) or RPMI-1640 medium (Sigma) at 30 °C with agitation at 180 rpm as stated. The pH of growth media was adjusted to 4.0 or 8.0 as required. *C. albicans* strains used in this study are listed in Supplementary Table S1. *C. albicans* clinical isolates used in this study were obtained as anonymised voice prosthesis isolated strains from microbiology services, East Kent Hospital University Foundation Trust.

### Cell growth assays

*C. albicans* cultures were grown overnight in YPD at 30 °C and then diluted to an OD_600_ of 0.1 in YPD or RPMI media with or without NaB at concentrations between 1 and 400 mM as stated. Growth was monitored in 96-well plates at 37 °C using a BMG Labtech FLUOstar plate reader over 24 h. The area under each growth curve (AUC) was calculated using GraphPad Prism. All experiments were performed in biological triplicate.

### Biofilm assays

One hundred microliter of 50% donor bovine serum (DBS) was added to each well of a 96-well plate and incubated with for 30 min at 30 °C. The plates were then washed twice with 100 μL of PBS buffer to remove excess DBS. Biofilms were established on serum-coated 96-well plates by inoculating with 1 × 10^6^ CFU/ml cells for 90 min at 37 °C without shaking to allow cell attachment. Wells were then washed twice with 100 μL PBS to remove any unattached cells, and RPMI-1640 media containing NaB supplemented between 50 and 400 mM. The plates were then incubated at 37 °C without shaking for 24 h.

Biofilm metabolic activity was quantified using an XTT assay (XTT Assay Kit ab232856, Abcam). Biofilms grown for 24 h were washed twice with 100 μL of PBS to remove any planktonic cells before proceeding to quantification. After washing, 100 μL of PBS was added to the wells along with 30 μg/mL XTT labelling reagent. and incubated at 37 °C for 4 h before absorbance measurements taken at 492 nm using a BMG LABTECH FLUOstar Omega plate reader. All experiments were performed in biological triplicates.

Biofilm biomass was estimated using crystal violet staining. Biofilms grown for 24 h were washed twice with 100 μL of PBS to remove planktonic cells before 100 μL of 0.1% crystal violet was added and incubated at room temperature for 15 min. Wells were washed four times with 100 μL of sterile water before leaving the plates to dry for 24 h. One hundred microliter of 30% acetic acid was added to each well and incubated for 15 min to release the stain. The plate was then spun down at 4,000 rpm for 5 min and supernatant transferred to a clean 96 well plate. The absorbance of the (550 nm) solution was then read using a BMG LABTECH FLUOstar Omega plate reader. All experiments were performed in biological triplicate.

### Hyphal formation assay

*C. albicans* were grown in YPD overnight at 30 °C with shaking at 180 rpm. The cultures were then diluted to an OD_600_ of 0.5 in RPMI ± 200 mM NaB supplementation and incubated at 37 °C at 180 rpm. Calcofluor white stain (Sigma) was added to the samples and images were captured at 2, 4, and 6 h following transfer to RPMI media using an Olympus IX81 fluorescence microscope using either DAPI filter set (350/465 excitation/emission) or using DIC at 100× magnification using an Andor Xyla 4.2 CMOS digital camera. All experiments were performed in biological triplicate.

### Western blotting

*C. albicans* were grown in YPD overnight at 30 °C with shaking at 180 rpm, sub-cultured and grown to an OD_600_ of 0.6 before adding 200 mM NaB and incubating at 37 °C at 180 rpm. 1 × 10^8^ cells were sampled after 0 min, 15 min, 30 min, and 60 min of NaB exposure and exposed to 200 μL of lysis buffer (1 mL of 1 M NaOH; 1 mL of 0.5 M EDTA pH8; 2 mL of 10% SDS; 200 μL ß-mercaptoethanol). The lysates were heated for 10 min at 90 °C before 5 μL of 4 M Acetic acid was added to each sample. Lysates were vortexed for 30 s before incubating them for 10 min at 90 °C. Fifty microliter of loading buffer (50% glycerol, 0.25 M Tris–HCl pH 6.8, 0.05% bromophenol blue) was added to each sample. An anti-rabbit Histone H4 primary antibody (Biorad AHP418, 1/800 dilution) was used in conjunction with an anti-rabbit Pgk1 primary antibody (a kind gift from the lab of Prof Mick Tuite, University of Kent, 1/10000) loading control to normalise for signal intensity. An anti-rabbit HRP labelled secondary antibody (Sigma 12–348, 1:10000) was used for ECL detection using a Syngene G-box Chemi XX6. The experiment was conducted in biological triplicate.

### Cell death assays and ROS detection

*C. albicans* were grown in YPD overnight at 30 °C with shaking at 180 rpm and used to inoculate RPMI to an OD600 nm of 0.5 at the stated pH and NaB additions for the indicated time. H_2_DCF-DA was added to a concentration of 5 μg/mL before incubating at 30 °C with constant shaking at 180 rpm for 1 h. Cultures were then washed three times in PBS and resuspended in PBS containing 2 μg/mL propidium iodide (PI) stain. DCF fluorescence intensity was analysed using a BD Accuri C6 Plus Personal Flow Cytometer (BD Biosciences). The FL1 (533/30 nm) filter was used to detect DCF fluorescence and the FL2 (585/40 nm) filter for propidium iodide. Data from 25,000 events were collected per sample and all experiments were carried out in biological triplicate. An *S. cerevisiae* strain lacking *COX4* (CGY638 *Mata his3Δ1 leu2Δ met15Δ ura3Δ Δcox4: HIS*) was used as positive ROS control (Leadsham et al., 2013), and a heat-killed (80 °C for 2 min) *C. albicans* culture sample was used as a positive PI control for evaluation of necrosis.

### Mitochondrial staining using MitoTracker Green FM

*C. albicans* cells were grown in YPD overnight at 30 °C with shaking at 180 rpm and cells were resuspended at 1 × 10^6^ cells/ml in appropriate pH adjusted media and incubated with 200 mM NaB for 1 h. Cells were then washed with PBS three times before incubating the cultures at room temperature for 30 min with 100 nM of Mitotracker Green FM. Cells were washed with 1 x PBS before capturing images using an Olympus IX81 fluorescence microscope using GFP filter set (395/509 excitation/emission) at 100x magnification using an Andor Xyla 4.2 CMOS digital camera.

### Mitochondrial matrix pH measurement using mtGFP

A *C. albicans* strain expressing GFP targeted to the mitochondrial matrix, mtGFP (Duvenage et al., 2019) was grown overnight in YPD at 30 °C. Cells were sub-cultured in YPD to an OD_600_ nm of 0.2 in a white 96 well plate with or without 200 mM NaB and/or 2 μM Antimycin A. Readings were taken every 5 min with shaking at 37 °C in an BMG Labtech OMEGA automatic plate reader using 488 nm excitation and 520 nm emission filters.

### Oxygen consumption assay using high resolution respirometry

*C. albicans* were grown overnight in YPD media and sub-cultured in pH adjusted RPMI medium to an OD_600_ nm of 1 × 10^6^ cells/ml. Cells were then added to the chamber of an Oroborus O2K Oxygraph High Resolution Respirometer. After obtaining a stable routine respiration level 200 mM NaB was added as either a single addition or as a 20 mM titration up to a final concentration of 200 mM. For 20 mM titration a 5 min interval between the additions of each drug was left to facilitate the detection of a full drug response and a reading taken following each addition (R1 to R10). Two micromolar Antimycin A (Ant A) (Sigma-Aldrich) was added after NaB addition was complete to assess the non-mitochondrial oxygen consumption (NMR). Data were acquired and analysed using Oroborus Datlab 4 software. All experiments were carried in biological triplicate.

### Intracellular calcium measurements

*C. albicans* expressing aequorin from an integrated plasmid (Sanglard, 2021) were grown to an OD_600_ nm of 0.5 and re-suspended at a density of 1×10^8^ cells/ml in fresh pH-adjusted medium. The substrate Coelenterazine (Invitrogen, Thermo Fisher) was then added to a final concentration of 5 μM. As a negative control, cells were treated with the equivalent volume of solvent (methanol). The samples were incubated in the dark for 5 h at 30 °C before being collected by centrifuging at 5,000 rpm for 5 min. The samples were then washed three times with PBS, resuspended in 200 μL of medium, and incubated for 30 min in the dark at 30 °C to allow for reconstitution of functional aequorin within cells. After the incubation, cells were transferred to a 96 well microplate and a luminescence reading was taken every 1 min, with 40 s shaking in between each read using a BMG Labtech OMEGA plate reader with a gain adjusted to 3,600. After 10 cycles to record the baseline luminescence, the plate was ejected and 200 mM NaB or 5 mM H_2_O_2_ were added. The plate was then returned to the plate reader and readings taken for a further 1 h. All experiments were conducted in biological triplicate.

### Cell viability assays

Wild type, catalase overexpression or catalase deletion *C. albicans* strains (a kind gifts from Professor Janet Quinn, University of Newcastle) were grown in YPD overnight at 30 °C with shaking at 180 rpm and diluted to an OD_600_ nm of 0.5 in RPMI. Two hundred millimetre NaB was added to cells with or without the presence of 50 μM BAPTA-AM. The pH was adjusted to either 4.0 or 8.0 before incubating the cells for 1 h at 37 °C at constant shaking at 180 rpm. The number of cells in a culture was counted, and 300 cells were plated to assess colony forming units (CFU) under each condition. The plates were incubated at 30 °C for 48 h prior counting colonies and calculating percentage viability. To assess the effects of NaB on *C. albicans* clinical isolates 1–4 over a range of pH values cells were grown overnight in YPD at 30 °C and sub cultured to an OD_600_ nm of 0.1 in YPD at pH 3, 4, 5, 6, 7, or 8 with or without 200 mM NaB and incubated at 37 °C with shaking for 2 h. Cells were then transferred to fresh YPD at the equivalent pH and incubated for 24 h at 37 °C before growth was assessed (OD_600_ nm). Experiments were conducted in biological quadruplicate.

## Results

### NaB inhibits growth and biofilm formation in a dose dependent manner and acts as an HDAC inhibitor in *Candida albicans*

**Figure 1:**
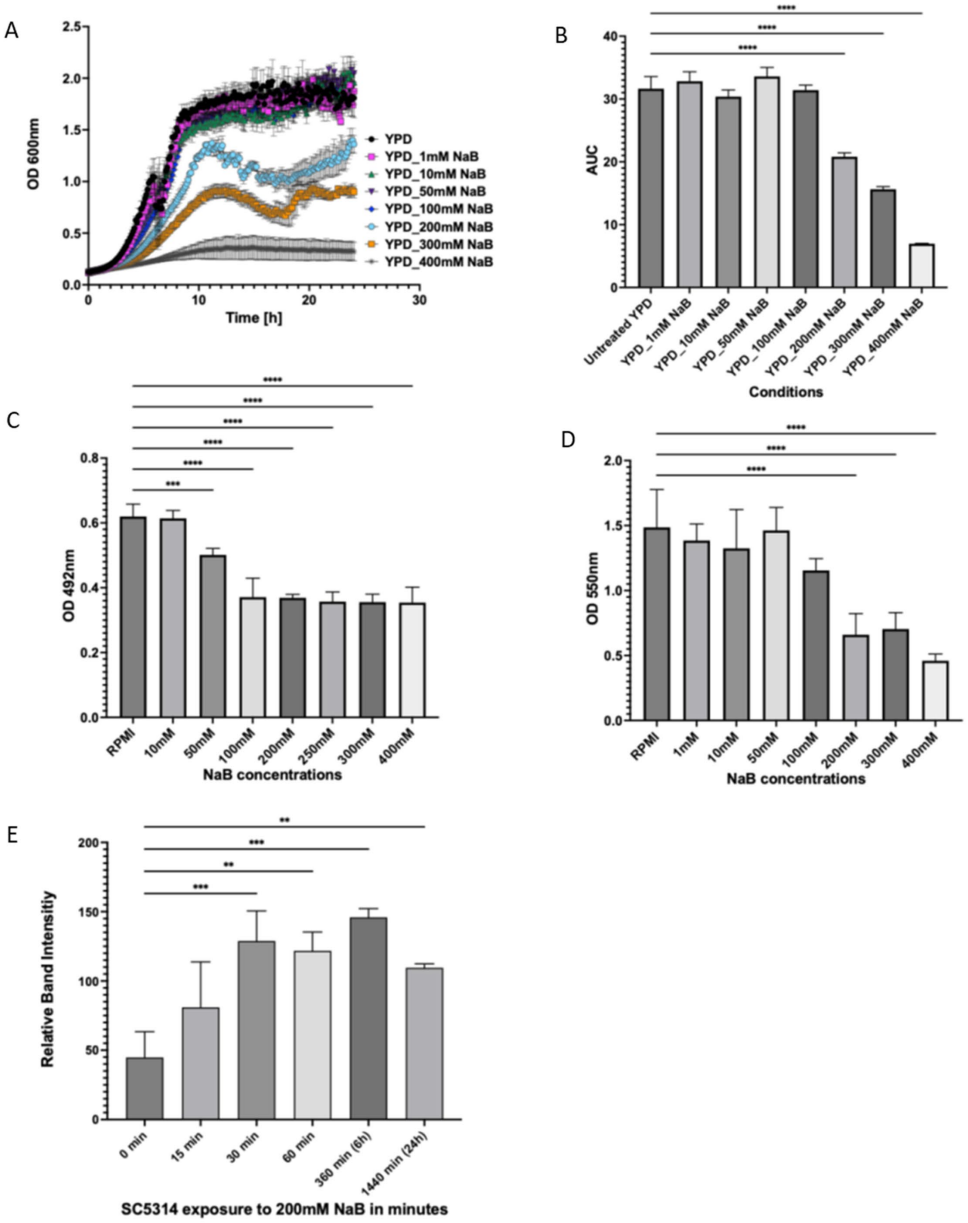
NaB acts to inhibit *C. albicans* growth, biofilm formation and acts as an HDAC inhibitor. *C. albicans* were grown for 24 h in YPD supplemented with various NaB concentrations **(A)** and the area under the curve (AUC) calculated as a proxy for overall growth during this time **(B)**. *C. albicans* were grown as biofilms for 24 h with NaB supplementation after cell attachment and quantified using either an XTT **(C)** or crystal violet **(D)** assay. *C. albicans* cells were grown to log phase and incubated with 200 mM NaB before total protein was extracted at 0 min, 15 min, 30 min, 60 min, 6 h, and 24 h. Western blot analysis of extracted protein was performed to detect the change in Histone H4 acetylation and plotted relative to a Pgk1 loading control **(E)**. The data shown are a representative of an average of three biological repeats. The error bars display the standard deviation. A one-way ANOVA using a Tukey multiple comparison test was used to determine statistical significance, *p*-value < 0.05. **p < 0.005, ***p < 0.0005, ****p < 0.00005.

We wished to characterise the effects of NaB on *C. albicans* growth and biofilm formation over a range of concentrations. The addition of NaB at concentrations between 200 mM and 400 mM significantly decreased *C. albicans* growth in planktonic culture (Figures 1A,B). Biofilm formation analysis using XTT assays showed a clear reduction in metabolic activity (Figure 1B) when growth media was supplemented with NaB between 50 mM and 400 mM (Figure 1C). Interestingly analysis using crystal violet staining showed a reduction in biofilm mass when NaB was supplemented, but only at concentrations above 200 mM (Figure 1D). These data suggest that the effects of NaB on growth and biofilm matrix production may be separable.

Since NaB has been shown to act as an HDAC inhibitor in several systems, we investigated whether the same is true in *C. albicans* at the concentration of 200 mM that we had found to significantly affect cell growth. Proteins were extracted after 0 min, 15 min, 30 min, 60 min, 6 h, and 24 h following 200 mM NaB exposure and histone H4 acetylation assessed by western blotting. A rapid accumulation of histone H4 acetylation could be observed upon 200 mM NaB exposure after 30 min and levels were maintained throughout the 24 h time course of the assay (Figure 1E).

### NaB addition affects respiration in a dose dependent manner

**Figure 2:**
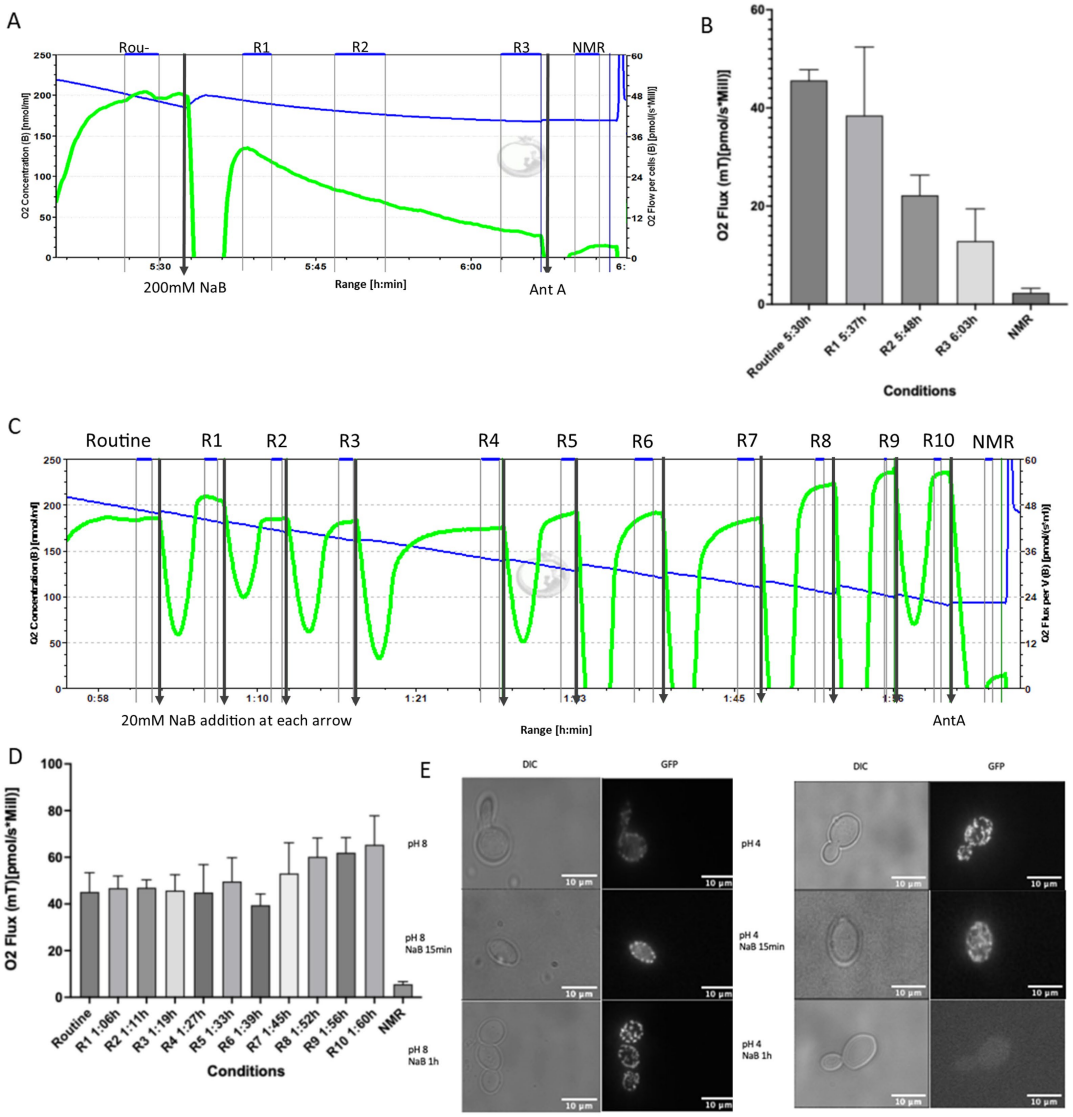
NaB modulates *C. albicans* mitochondrial respiration in a dose dependent manner. **(A)** Respirometry profile was obtained for *C. albicans* cells following addition of a single dose of 200 mM NaB. **(A)** The oxygen flux per cell (pmols/s*Mill) is shown as the green line, whereas the blue line represents the oxygen concentration. The routine phase indicates the steady state oxygen flux before NaB addition. Oxygen flux was measured at routine (R), and three time points following 200 mM NaB addition (R1, R2, and R3) as well as following addition of 2 μM Antimycin A to assess non-mitochondrial respiration (NMR) **(A,B)**. The same experiment was conducted, but this time NaB was added in 20 mM doses up to a final concentration of 200 mM, and oxygen flux measured after each NaB addition (R1–R10) **(C,D)**. The data shown represents an average of three biological repeats, and the error bars indicate the standard deviation. **(E)** DIC and GFP fluorescence microscopy were used to examine the effects on mitochondrial morphology following addition of 200 mM NaB to *C. albicans* cells cultured in pH 8 or pH 4 RPMI media. Cells were visualised at x100 magnification. The scale bar is 10 μm.

*C. albicans* is a Crabtree negative yeast and so relies on respiration for effective growth (Duvenage et al., 2019). We therefore examined the effects of NaB addition on respiration as either a single 200 mM dose, or as a titration of 20 mM additions up to 200 mM. Similar doses of NaB have been used as a dietary supplement with measurable therapeutic effects (Luceri et al., 2016). The application of 200 mM NaB as a single dose led to rapid decline in respiration within 30 min of addition (Figures 2A,B). The subsequent addition of Antimycin A, which blocks electron transport at complex III of the electron transport chain, led to a further decrease in oxygen consumption indicating significant but incomplete inhibition. In contrast to the effects of acute administration, NaB titration led to an increase in respiration over the duration of the experiment. The subsequent addition of Antimycin A led to a rapid decrease in respiration indicating that the increase in oxygen consumption observed upon NaB titration was caused by elevated electron transport chain activity (Figures 2C,D). These data suggest that although NaB has clear effects on *C. albicans* mitochondrial respiration, this can result in either inhibition or enhancement depending on the level and frequency of NaB exposure.

As the pH varies within different areas of the gut, we examined whether the addition of 200 mM NaB at pH 4.0 and pH 8.0 led to different effects on mitochondrial morphology and membrane potential using Mitotracker Green. At pH 8.0 untreated dividing yeast cells displayed the expected mixture of tubular, fused, and fragmented mitochondria (Figure 2E). A more fragmented appearance was observed after 1 h of 200 mM NaB treatment, indicating mitochondrial stress (Figure 2E). At pH 4.0 mitochondrial fragmentation could be observed in both untreated and NaB-treated cells (Figure 2E). Surprisingly, after 1 h of treatment with 200 mM NaB incubation at pH 4.0 the mitotracker green signal could no longer be visualised (Figure 2E). As mitotracker green requires the presence of a membrane potential to accumulate in mitochondrial membranes the loss of signal may indicate that NaB treatment leads to depolarisation of the mitochondrial inner membrane potential, which is commonly linked to the accumulation of ROS (Zhao et al., 2019).

### The loss of Set3 leads to an insensitivity to HDAC inhibition and the effects of NaB on biofilm growth

**Figure 3:**
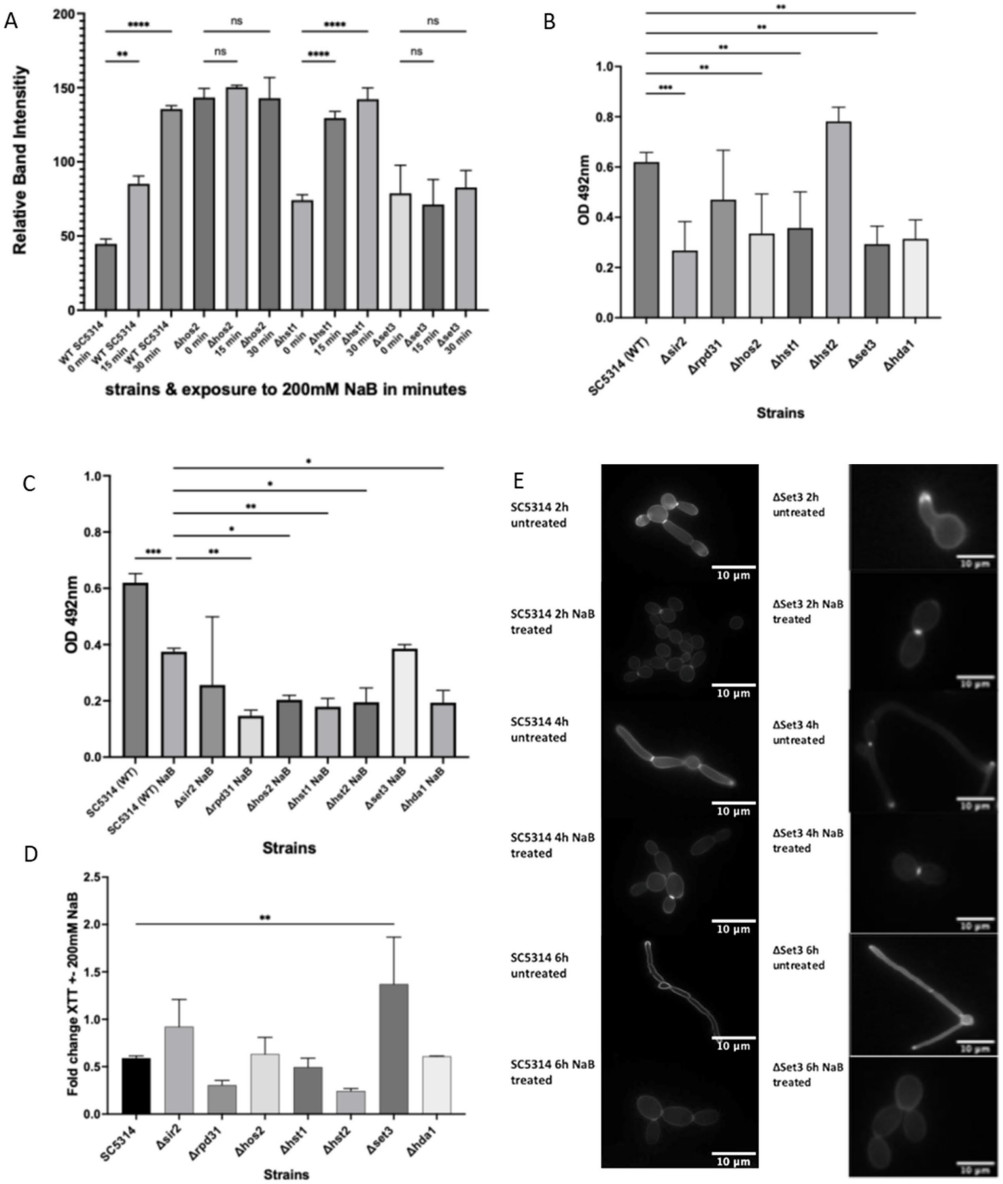
NaB has Set3 dependent and independent modes of action. *C. albicans* cells were grown to log phase and subjected to 200 mM NaB, total protein was extracted at 0 min, 15 min, and 30 min and a western blot carried out to detect changes in histone H4 acetylation relative to Pgk1 loading control **(A)** and Supplementary Figure S1. Wild type *C. albicans* or strains containing deletions in selected HDAC genes were grown as biofilms in the absence **(B)** or presence **(C)** of 200 mM NaB for 24 h and assessed using an XTT assay. The fold change of NaB treated/untreated biofilms as assessed by XTT reading were calculated **(D)**. The data shown are a representative of an average of three biological repeats. The error bars display the standard deviation. A one-way ANOVA using a Tukey multiple comparison test was used to determine statistical significance, *p*-value < 0.05. *C. albicans* wild type or *Δset3* cells were grown in RPMI media at 37 °C to induce filamentation in the presence or absence of 200 mM NaB, stained with calcofluor white and images taken at 2 h, 4 h, and 6 h **(E)**. Cells were visualised at x100 magnification under DAPI illumination (360 nm/460 nm Ex/Em). Sale bar = 10 μm. *p < 0.05, **p < 0.005, ***p < 0.0005, ****p < 0.00005.

As we had observed an increase in H4 acetylation upon NaB treatment we wished to determine whether loss of any particular HDAC gene led to measurable changes in this response. We investigated whether the pattern of HDAC activity observed upon deletion of either loss of genes representing class I, class III HDACs and the Set3 complex (*SET3*, *HST1* or *HOS2*) was observed in response to NaB treatment. A western blot was carried out on protein extracts taken after 0 min, 15 min, and 30 min of 200 mM NaB exposure. A rapid accumulation of histone H4 acetylation could be observed upon NaB exposure after 15 min and increased further after 30 min NaB exposure in *Δhst1* as had been observed in wild type cells (Figure 3A). In contrast no significant increase in H4 acetylation could be seen in *Δset3* cells within 30 min of NaB exposure (Figure 3A). Elevated histone H4 acetylation levels could be observed in *Δhos2* at all time points and no further increase could be detected upon NaB exposure (Figure 3A). Having observed that loss of single HDAC genes could affect the response to NaB exposure we decided to examine the effects of a wider range of candidates. To test this we examined the effects the following deletions on biofilm growth: *Δsir2* (Class III; part of sirtuin family, NAD^+^ dependent HDAC), *Δrpd31* (Class I; necessary to initiate filamentous growth), *Δhos2* (Class I; part of the Set3 complex’ core; essential for Set3C assembly), *Δhst1* (Class III; members of sirtuin family, part of the peripheral part of the Set3 HDAC complex), *Δhst2* (Class III), *Δset3* (Class III; Set3 is an NAD^+^ dependent HDAC, important for morphogenesis), *Δhda1* (Class II). Set3, Hst1 and Hos2 are HDACs found within the seven-member complex Set3C complex. Set3, Hos2, Snt1 and Sif2 form part of the core and are crucial for the assembly of the complex, whilst Hos4, Hst1 and Cpr1 are peripheral members. The Set3C complex has been directly linked to the regulation of the biofilm regulatory network as it can control the activity of five of the six core biofilm regulatory transcription factors *NRG1, BRG1, TEC1, NDT80*, and *ROB1* (Garnaud et al., 2016). In addition Set3C can control the expression of, Nrg1, which is involved in the regulation of cellular dispersion from biofilms (Uppuluri et al., 2010).

A clear reduction in metabolic activity could be observed upon 200 mM NaB exposure when *SIR2, HOS2, HST1, SET3*, or HDA*1* were deleted compared to the wild type. However, a decrease was not observed in *RPD31* or HST*2* deletion strains (Figure 3B). In NaB treated biofilms reduced metabolic activity could be detected in *Δrpd31*, *Δhos2*, *Δhst1*, *Δhst2*, and *Δhda1*, but not in *Δsir2* or *Δset3* strains when compared to the wild type (Figure 3C). Fold change analysis showed that in all strains, with the notable exception of *Δset3*, the addition of 200 mM NaB led to relative reduction in metabolic activity that was equivalent to that observed in wild type (Figure 3D).

As the deletion of *SET3* led to a loss of both NaB induced H4 acetylation and relative biofilm growth reduction we considered whether Set3 inhibition may provide a mechanism for the observed phenotypes. We therefore investigated whether the loss of *SET3* affected the previously reported ability of NaB treatment to inhibit hyphal formation in *C. albicans* (Nguyen et al., 2011). We examined hyphal growth of NaB treated and non-treated *SET3* deletions strains over a time course of 2–6 h following transfer to RPMI, a media that effectively induces hyphal transition. Hyphae were clearly visible after 2 h of growth in RPMI, and as long extension after 6 h in both untreated wild type and *∆set3* cells (Figure 3E). Hyphal induction was inhibited in both wild type and *∆set3* treated with NaB. This suggests that the Set3 dependent effects of NaB on HDAC inhibition and metabolic activity within biofilms can be separated from its effects on hyphal induction (Figure 3E).

### NaB addition leads to loss of viability under acidic conditions, ROS production and sensitivity to peroxide and calcium stress

**Figure 4:**
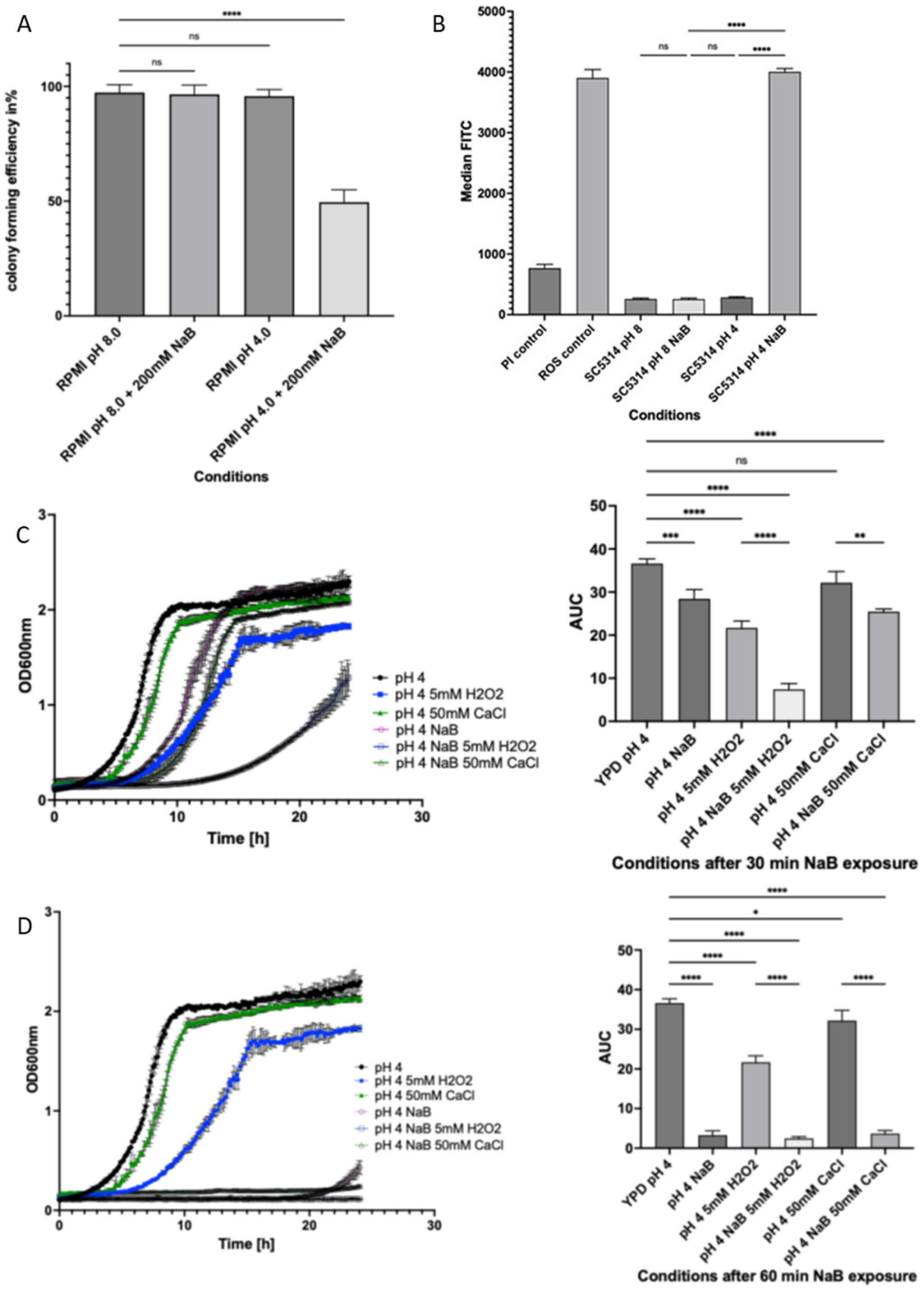
Acidic pH enhances the antifungal effects of NaB against *C. albicans. C. albicans* were grown to log phase and the effects of 200 mM treatment at pH 4.0 or 8.0 were assessed by a colony forming unit (CFU) assay **(A)**. Flow cytometry was used to assess ROS levels following 200 mM treatment at pH 4.0 or 8.0 by H_2_DCF-DA fluorescence using a FITC filter set. A known high ROS producing strain lacking the *COX4* gene was used as a ROS positive control **(B)**. The effects of 200 mM NaB exposure for either 30 min **(C)** or 60 min **(D)** on C. albicans ability to cope with exogenous 5 mM H2O2 or 50 mM CaCl2 stress was tested by a growth assay and the area under the curve (AUC) used to allow for growth comparison. The data shown are a representative of an average of three biological repeats. The error bars display the standard deviation. A one-way ANOVA using a Tukey multiple comparison test was used to determine statistical significance, *p*-value < 0.05. *p < 0.05, **p < 0.005, ***p < 0.0005, ****p < 0.00005.

As we had observed that NaB treatment affected mitochondrial morphology in a pH dependent manner, we wished to determine its effects on viability and stress resistance. The exposure of wild type *C. albicans* cells to 200 mM NaB for 2 h led to a significant reduction in viability at pH 4.0 but did not affect viability at pH 8.0 (Figure 4A). To determine whether the same effects could be observed in other strains of *C. albicans* we exposed four clinical isolates to NaB at 200 mM at pH 3, 4, 5, 6, 7, 8 for 2 h before replacing with media at the same pH lacking NaB and incubating overnight. All strains grew equally over the pH range as would be expected for *C. albicans* and the addition of 200 mM NaB at pH 6, 7, and 8 did not lead to a significant reduction in viability. This is consistent with the fungistatic we had observed in SC5314 wild type cells. As pH decreased, we saw a drop in viability with complete inhibition at pH 3 and 4, which is consistent with the fungicidal effects of NaB under acidic conditions (Supplementary Figure S2).

To investigate the antifungal mode of action further we examined ROS production using flow cytometry. Our analysis revealed that the addition of 200 mM NaB at pH 4.0 led to the accumulation of high levels of ROS, assessed by fluorescence output of the indicator dye H_2_DCF-DA. However, cells grown at pH 8.0 with or without NaB or untreated cells grown at pH 4.0 showed low ROS accumulation (Figure 4B). The co-staining of cells with propidium iodide showed that treatment of cells at either pH 8.0 or 4.0 was not associated with dye uptake, indicating that loss of viability associated with NaB treatment was not associated with necrosis (Figure 4B).

A loss of mitochondrial function has been linked to an increased sensitivity to oxidative stress in yeast (Grant et al., 1997; Bambach et al., 2009) and the accumulation of ROS has been linked to a loss in calcium regulation and overload within the matrix (Brookes et al., 2004). We therefore investigated if NaB treated cells grown at pH 4.0 were more sensitive to oxidative stress (H_2_O_2_) or exogenous addition of calcium. *C. albicans* cells were exposed to 200 mM NaB for 30 (Figure 4C) or 60 min (Figure 4D) at pH 4.0 prior to analysing their growth for 24 h in media containing either 5 mM H_2_O_2_ or 50 mM CaCl_2_. A significant decrease in growth and an increased lag phase could be observed in *C. albicans* when cells were pre-treated for 30 mins with 200 mM NaB pre-treatment at pH 4.0 when compared to the untreated control (Figure 4C). Almost no growth could be observed when 60 min NaB pre-treated cells were grown in media containing either 5 mM H_2_O_2_ or 50 mM CaCl_2_ at pH 4.0 compared to their untreated H_2_O_2_ or CaCl_2_ controls (Figure 4D).

### NaB induced *Candida albicans* cell death at pH 4.0 is driven by a loss of calcium homeostasis and ROS accumulation

The regulation of calcium homeostasis has been linked to mitochondrial function, reactive oxygen species (ROS) accumulation, and cell viability. We therefore investigated whether intracellular calcium homeostasis was perturbed in NaB treated *C. albicans* cells grown in RPMI at pH 8.0 and pH 4.0. To do this, we used the Aequorin system (Sanglard, 2021), which provides a fluorescence readout of cytosolic calcium levels. As a positive control 5 mM H_2_O_2_, previously reported to increase cytosolic calcium levels detected by the Aequorin protein, was included (Popa et al., 2010).

**Figure 5:**
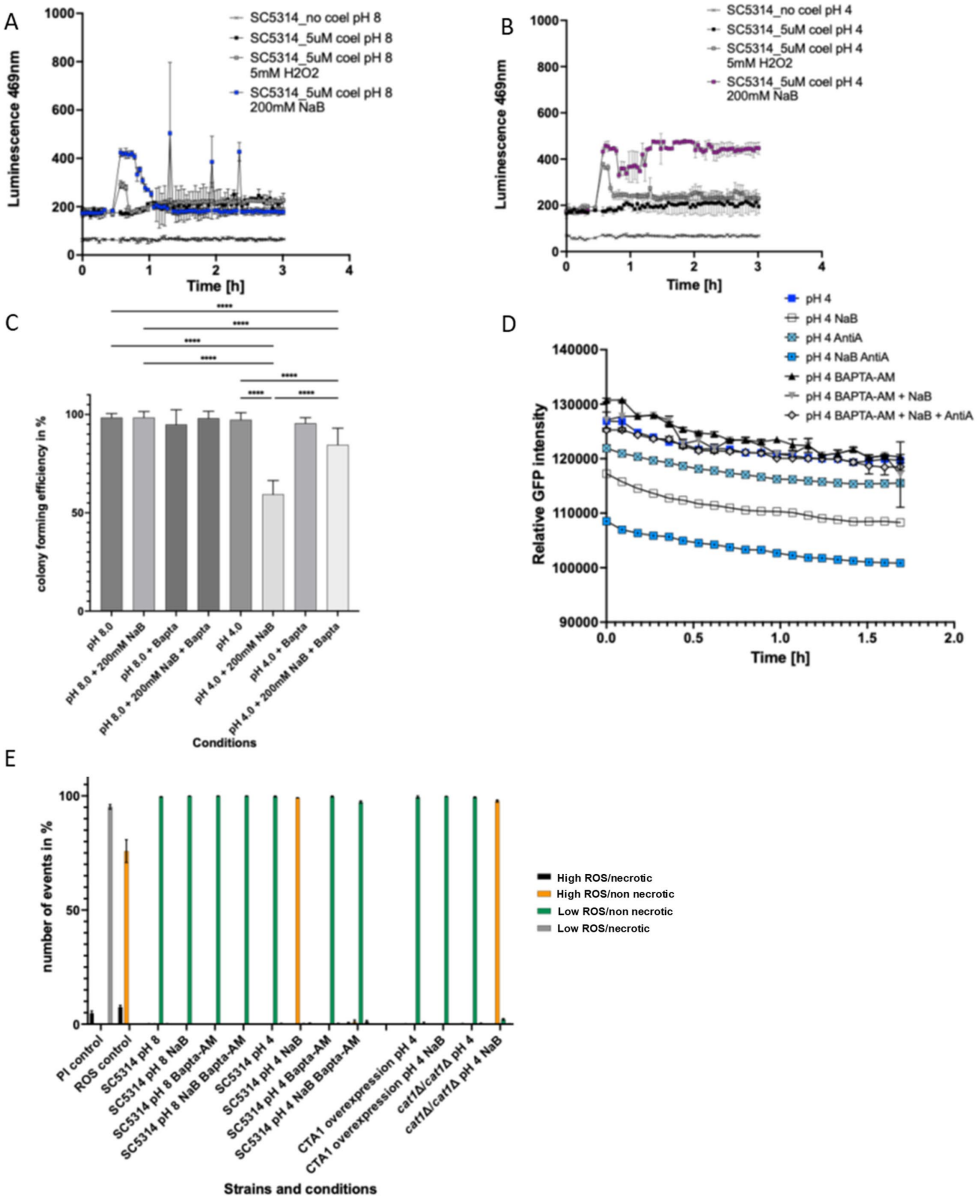
ROS elevation and loss of calcium homeostasis underlies NaB induced *C. albicans* cell death under acidic conditions. *C. albicans* were grown to log phase and the effects of 200 mM treatment at pH 4.0 or 8.0 alone or in addition to oxidative stresses (5 mM H_2_O_2_). The effects of this treatment were assessed using Aequorin luminescence output at 469 nm over a period of 3 h. **(A,B)** The effect of 200 mM NaB treatment for 2 h at pH 4.0 or 8.0 alone or in addition to 50 μM BAPTA-AM on viability was assessed by colony forming unit assay. **(C)** The effects of 200 mM NaB treatment for 2 h at pH 4.0 alone or in addition to 50 μM BAPTA-AM on mitochondria matrix targeted GFP fluorescence was assessed over a period of 1.75 h. The addition of 2 μM antimycin A was used as a positive control. **(D)** ROS accumulation was assessed by H_2_DCF-DA and necrosis (PI uptake) assessed using flow cytometry on *C. albicans* cells treated with 200 mM NaB at pH 4.0 or 8.0 alone or in addition to 50 μM BAPTA-AM or in a strain overexpressing catalase (CTA1) or lacking CTA1. Data are represented as the % of cells within the population that had either low or high ROS/PI signal as defined by established gates. **(E)** The data shown are a representative of an average of three biological repeats. The error bars display the standard deviation. A ne-way ANOVA using a Tukey multiple comparison test was used to determine statistical significance, *p*-value < 0.05. ****p < 0.00005.

As expected, the addition of 5 mM H_2_O_2_ led to a transient spike in intracellular calcium at both pH 8.0 and 4.0 (Figures 5A,B) in wild type *C. albicans* cells which returned to baseline levels within a few minutes (Figures 5A,B). When 200 mM NaB was added at pH 8.0, a larger spike of increased intracellular calcium was observed than upon 5 mM H_2_O_2_ addition, but this too returned to baseline levels after a few minutes (Figure 5A). Interestingly, over the time course of 3 h, several smaller calcium spikes appeared which may indicate that the cells still experienced smaller bursts of increased intracellular calcium after NaB treatment (Figure 5A). In contrast, when 200 mM NaB was added at pH 4.0, intracellular calcium levels rose but failed to return to baseline over the course of the assay, indicating a loss of homeostasis (Figure 5B).

Given that 200 mM NaB treatment caused both calcium imbalance and loss of viability at pH 4.0 we tested whether buffering intracellular calcium using the calcium chelator, BAPTA-AM, could rescue these phenotypes. Treatment with BAPTA-AM (50 μM) partially restored CFU values, to 85%, when wild type *C. albicans* cells were exposed to 200 mM NaB at pH 4.0 (Figure 5C). We observed a loss of Mitotracker Green signal when 200 mM NaB was added to cells at pH 4.0, which may indicate a loss of membrane potential. The rapid loss of mitochondrial membrane potential can lead to acidification of the matrix as a result protons leaking from the intermembrane space into the matrix (Hawrysh and Buck, 2019). GFP can be used an intracellular pH sensor (Kneen et al., 1998) and so the fluorescence output of a mitochondrial matrix targeted GFP was monitored to report the effects of both 200 mM addition and BAPTA-AM co-addition at pH 4.0. The addition of either 200 mM NaB or the complex III inhibitor Antimycin A led to decline in GFP signal over the time of the experiment, consistent with a loss of membrane potential and acidification of the matrix. Consistently, the addition of BAPTA-AM restored the fluorescent signal of mtGFP to levels of untreated cells suggesting that preventing the accumulation of calcium could also prevent the loss of membrane potential and mitochondrial matrix acidification (Figure 5D).

Finally, we examined the relationship between calcium, ROS, and antioxidant capacity. As shown previously the addition of 200 mM NaB at pH 4.0 resulted in ROS accumulation that was not accompanied by necrosis (Figure 5E; Supplementary Figure S3). Co-treatment of wild type *C. albicans* cells with 50 μM BAPTA-AM and 200 mM NaB at pH 4.0 prevented ROS accumulation (Figure 5E; Supplementary Figure S3). The accumulation of ROS observed upon treatment with 200 mM NaB at pH 4.0 was prevented when catalase, *CTA1*, was overexpressed, but was not in a strain deleted for *CTA1*, suggesting a role for catalase-mediated detoxification.

## Discussion

The human gastrointestinal tract is a continuum of pH values ranging from highly acidic in the stomach to near neutral in the colon. This pH gradient partition microbial niches and metabolic activity (Duncan et al., 2009; Yamamura et al., 2023). SCFAs are produced in the colon through fermentation of dietary fibres and saccharides by bacteria causing weakly acidic conditions in the intestinal tract. In acidic conditions, butyrate is mainly found as its protonated form butyric acid whilst a small proportion will be present as butyrate ion (conjugated base). In the colon, where SCFAs accumulate, acidic microenvironments may potentiate butyrate’s antifungal effects. Physiological concentrations of butyrate in the human colon are reported to exist within the millimolar range. Faecal and luminal measurements of butyrate have been recoded between 10 and 25 mM, however this will vary depending on diet and fermentative substrate availability (Cummings et al., 1987; Gasaly et al., 2021). These values indicate that luminal butyrate concentrations approaching 200 mM are unlikely to reflect normal physiological conditions. However, several studies demonstrate that such high levels of butyrate administration are achievable and tolerated within mammalian systems. For example in murine models levels of sodium butyrate administration at and above 200 mM were well tolerated and showed beneficial effects within the intestinal tract (Shah and Cowles, 2021; Yu et al., 2023). In a human clinical study, butyrate administration above 200 mM was also reported as well tolerated and associated with beneficial clinical outcomes (Luceri et al., 2016). Collectively, these findings suggest that while 200 mM exceeds physiological human colonic levels, it may represent a feasible pharmacological or therapeutic level. Our findings demonstrate that the antifungal effects of butyrate on *Candida albicans* occur at levels that are only likely to be achieved by supplementation, or potentially in cases of localised fermentation, and are strongly influenced by pH. At pH 8.0 NaB acted fungistatically by suppressing growth, hyphal formation and biofilm development, potentially via histone deacetylase (HDAC) inhibition. By contrast, under acidic conditions (pH 4.0), NaB exhibited strong fungicidal activity suggesting that its effects as an antifungal may differ within different parts of the gut.

At neutral pH, butyrate predominantly exists as its conjugate base, which can inhibit HDACs (Davie, 2003; Sekhavat et al., 2007). We observed that NaB acts as a potent HDAC inhibitor which may provide a mechanism for its effects on cell growth and biofilm formation. Our results further implicate the Set3C complex as a potential target for NaB as the loss of *SET3* reduced cellular sensitivity to NaB and prevented the hyperacetylation of H4. This suggests that components of Set3C may serve as functional targets of NaB. Set3C controls multiple biofilm regulators, including *NRG1*, *TEC1*, and *NDT80* (Garnaud et al., 2016) and we observed that loss of Set3 led to a reduced effect of NaB upon inhibition of biofilm growth. However the acknowledged effects of NaB on preventing yeast-to-hyphae transition (Wurtele et al., 2010; Zacchi et al., 2010) was not altered upon *SET3* deletion, suggesting that there may be multiple effectors are affected by NaB activity that can account for its range of effects on cell behaviour.

**Figure 6:**
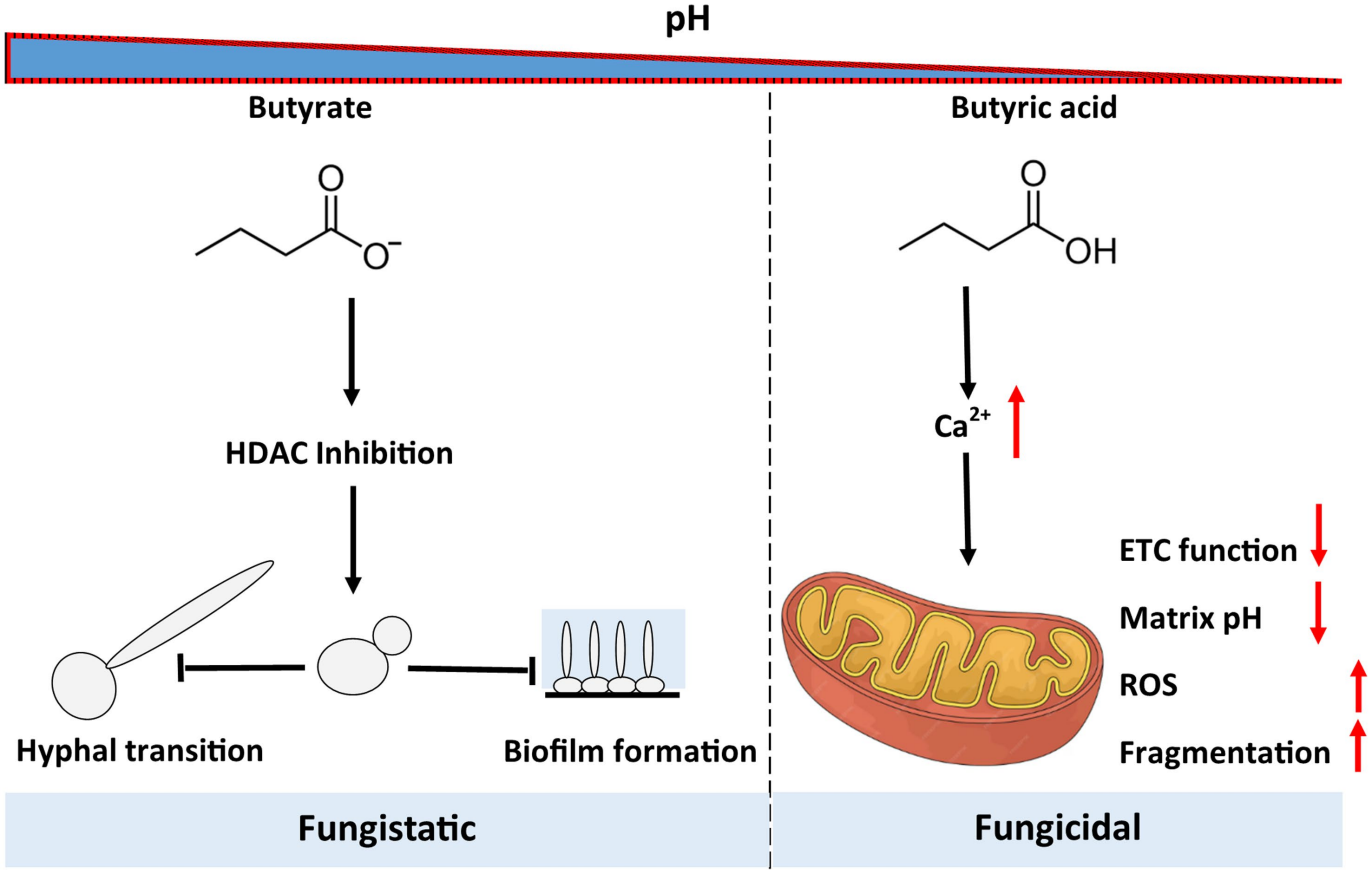
The pH dependent antifungal effects of NaB on *C. albicans.* The addition of high levels of NaB (200 mM) has a fungistatic effect at pH 8.0, inhibiting growth, biofilm formation, and hyphal transition, potentially via its action as an HDAC inhibitor. At the same concentration under acidic conditions where NaB exists in its protonated form, butyric acid, it has strong fungicidal effects that are triggered by a loss of calcium homeostasis and a resultant collapse of mitochondrial membrane potential, acidification of the mitochondrial matrix, and high levels of ROS production.

Under acidic condition, where butyrate is largely protonated (butyric acid), NaB exposure led to rapid cell death accompanied by mitochondrial depolarization, ROS accumulation, and loss of calcium homeostasis. In yeast calcium regulation (Carraro and Bernardi, 2016) and ROS production (Farrugia and Balzan, 2012) have been shown to be a crucial mediators of regulated cell death. We observed that an acute application of NaB was sufficient to inhibit electron transport chain function. The rapid inhibition of electron transport, such as by use of a potent bc1 inhibitor like Antimycin A, is commonly linked to elevated levels of ROS that can contribute to cell death (Ocampo et al., 2012). Our data also suggests that mitochondrial inhibition upon NaB treatment at pH 4.0 is dependent on loss of calcium homeostasis as this could be prevented by the addition of the calcium chelator BAPTA-AM. Given that calcium chelation could prevent acidification of the matrix and loss of viability we propose this as the primary driver of the observed mitochondrial dysfunction and subsequent ROS accumulation that act as further drivers of cell death (Figure 6).

Overall, we have shown that NaB displays pH-dependent antifungal activity against *C. albicans*. Its fungistatic effects at neutral pH are accompanied by an ability to prevent hyphal transition and reduce biofilm formation at high concentration (200 mM), as may be found upon consumption of certain foods or under butyrate supplementation. Clinical research groups could identify potential therapeutic use of butyrate in a wide spectrum of gastroenterological disorders to exploit its effects on inflammation, proliferation of intestinal epithelial cells, and regulation of fluid and electrolyte uptake in disorders like irritable bowel syndrome (Vanhoutvin et al., 2009), cholera (Rabbani et al., 1999), congenital chloride diarrhoea (Berni Canani et al., 2004; Wedenoja et al., 2008), prevention of colorectal cancer (Scharlau et al., 2009), and inflammatory bowel disease (Segain, 2000; Thibault et al., 2010). However, in the acidic compartments of the gut NaB can act in a fungicidal manner potentially via effects on mitochondria leading to ROS accumulation, and calcium dysregulation. These findings have implications for gut microbial ecology and diseases where reduced butyrate production and fungal overgrowth coincide.

## Data Availability

The raw data supporting the conclusions of this article will be made available by the authors, without undue reservation.
