# Supplementary material for: Investigating the pH dependent antifungal effects of butyrate on Candida albicans
Source: Front Microbiol. 2026 Mar 23;17:1793162. doi: 10.3389/fmicb.2026.1793162 (PMC13050787; doi:10.3389/fmicb.2026.1793162)
Supplement: Supplementary file 1 [file Table_1.DOCX]

**Table S1 – *C. albicans* strains used in this study**

| Strain Name | Genotype |
| --- | --- |
| *Δrpd31* | *rpd31Δ::C.d.HIS1/rpd31Δ::C.m.LEU2*  *arg4Δ/arg4Δ his1Δ/his1Δ leu2Δ/leu2Δ URA3/ura3Δ::λimm434 IRO1/iro1Δ::λimm434* |
| *Δsir2* | *ura3Δ::λimm434/ura3Δimm434 his1::hisG/his1::hisG arg4::hisG/arg4::hisG sir2::HIS1/sir2::ARG4* |
| *Δhos2* | *hos2Δ::C.d.HIS1/hos2Δ::C.m.LEU2*  *arg4Δ/arg4Δ his1Δ/his1Δ leu2Δ/leu2Δ URA3/ura3Δ::λimm434 IRO1/iro1Δ::λimm434* |
| *Δhst1* | *hst1Δ::C.d.HIS1/hst1Δ::C.m.LEU2*  *arg4Δ/arg4Δ his1Δ/his1Δ leu2Δ/leu2Δ URA3/ura3Δ::λimm434 IRO1/iro1Δ::λimm434* |
| *Δhst2* | *hst2Δ::C.d.HIS1/hst2Δ::C.m.LEU2*  *arg4Δ/arg4Δ his1Δ/his1Δ leu2Δ/leu2Δ URA3/ura3Δ::λimm434 IRO1/iro1Δ::λimm434* |
| *Δset3* | *arg4Δ/arg4Δ his1Δ/his1Δ leu2Δ/leu2Δ URA3/ura3Δ::λimm434 IRO1/iro1Δ::λimm434 set3Δ::C.d.HIS1/set3Δ::C.m.LEU2* |
| *Δhda1* | *ura3Δ::λimm434/ura3Δ::λimm434 his1::hisG/his1::hisG arg4::hisG/arg4::hisG hda1Δ::arg4/hda1Δ::his1* |
