# Supplementary figures and images for: Investigating the pH dependent antifungal effects of butyrate on Candida albicans
Source: Front Microbiol. 2026 Mar 23;17:1793162. doi: 10.3389/fmicb.2026.1793162 (PMC13050787; doi:10.3389/fmicb.2026.1793162)

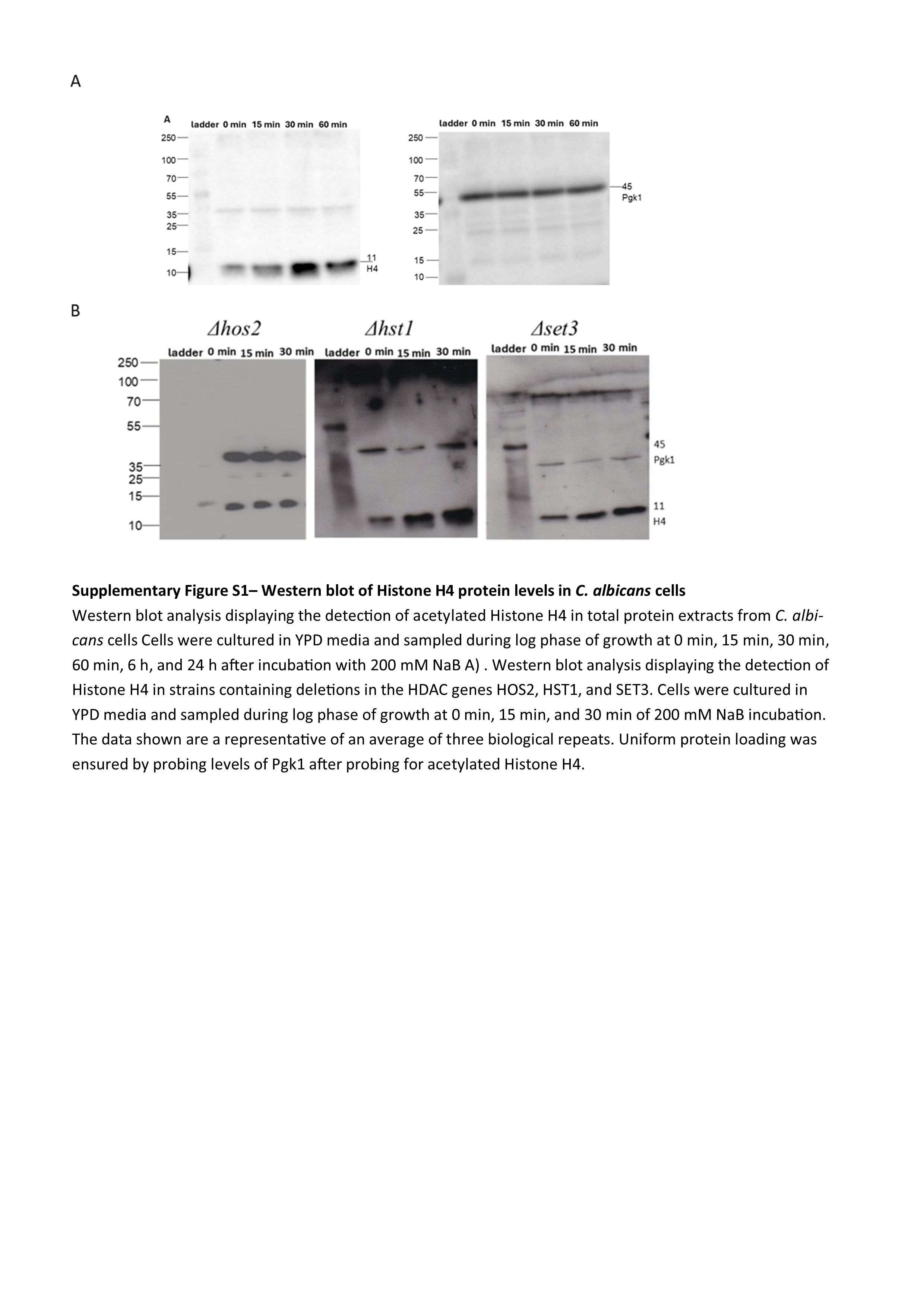

Supplement: Supplementary file 2 [file Image_1.TIF]

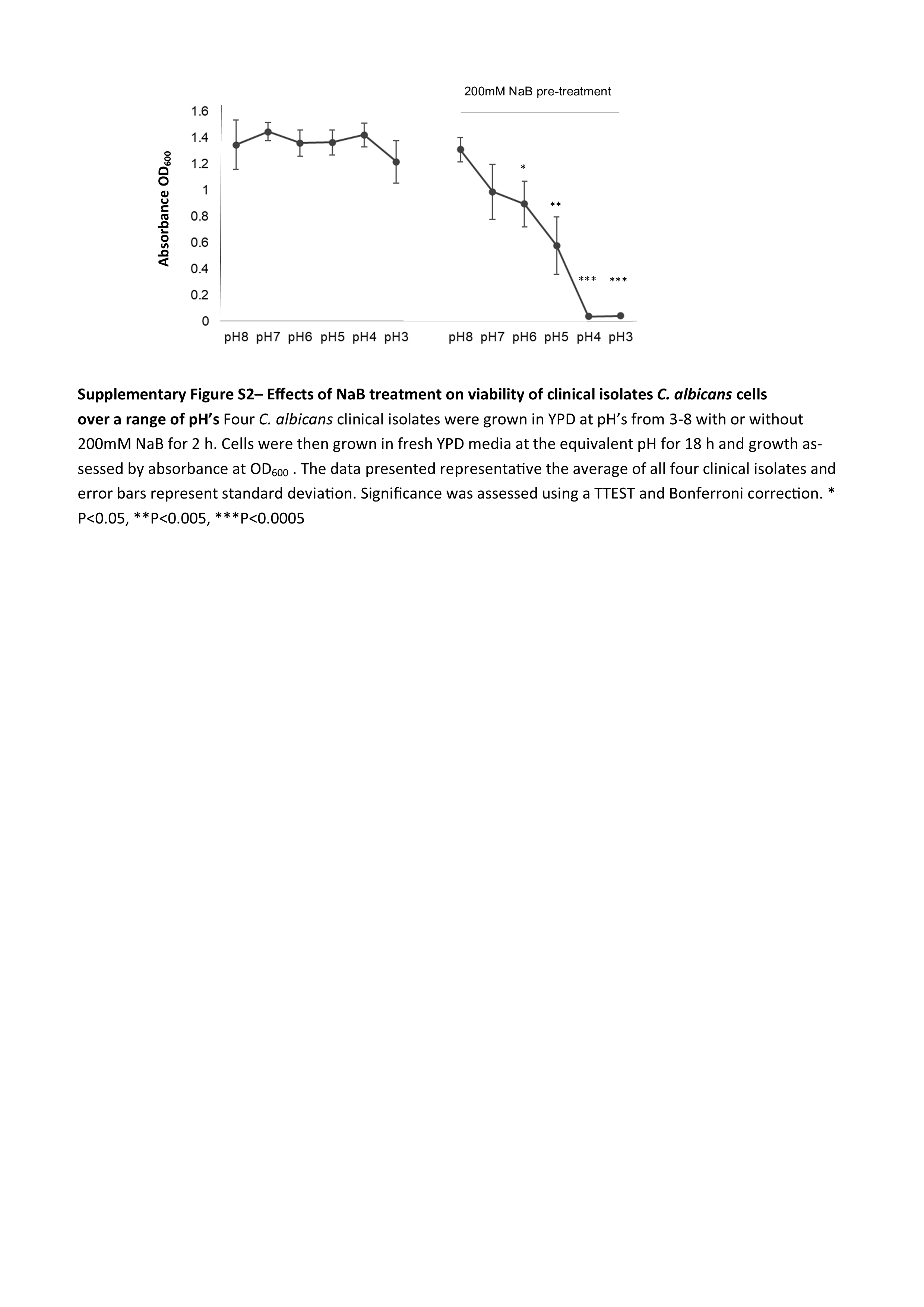

Supplement: Supplementary file 3 [file Image_2.TIF]

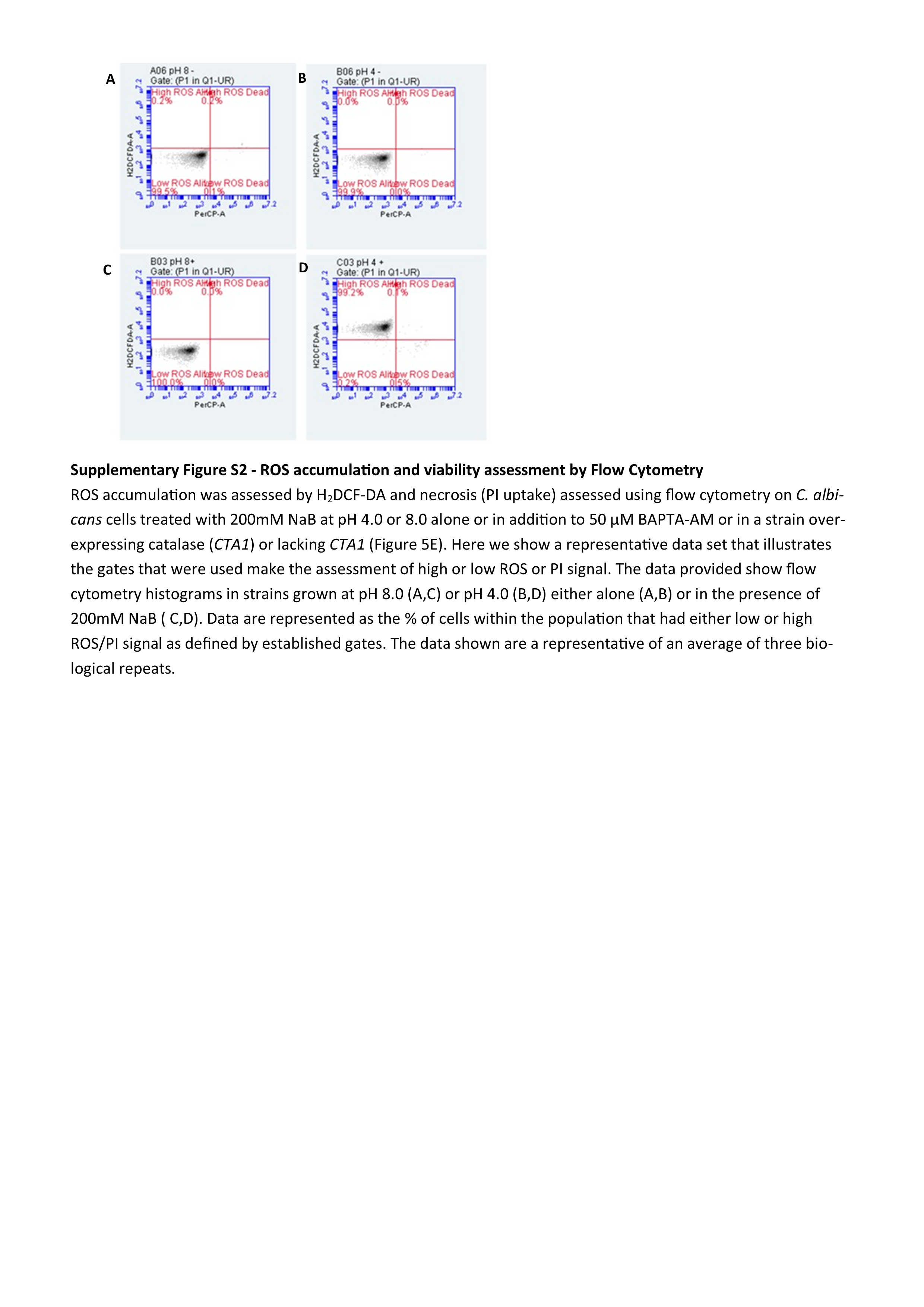

Supplement: Supplementary file 4 [file Image_3.TIF]
